# National stakeholders’ perceptions of the processes that inform the development of national clinical practice guidelines for primary healthcare in South Africa

**DOI:** 10.1186/s12961-018-0348-3

**Published:** 2018-07-31

**Authors:** Tamara Kredo, Sara Cooper, Amber Abrams, Karen Daniels, Jimmy Volmink, Salla Atkins

**Affiliations:** 1 Cochrane South Africa, South African Medical Research Council, Cape Town, South Africa; 2 Health Systems Research Unit, South African Medical Research Council, Cape Town, South Africa; 3 Health Policy and Systems Division, School of Public Health and Family Medicine, University of Cape Town, Cape Town, South Africa; 4 Centre for Evidence-Based Health Care, Faculty of Medicine and Health Sciences, Stellenbosch University, Cape Town, South Africa; 5 Deans Office, Faculty of Medicine and Health Sciences, Stellenbosch University, Cape Town, South Africa; 6 Department of Public Health Sciences, Karolinska Institutet, Stockholm, Sweden; 7 New Social Research and Faculty of Social Sciences, University of Tampere, Tampere, Finland

**Keywords:** Clinical practice guideline, Primary healthcare, Qualitative study, Guideline development

## Abstract

**Background:**

There is increased international focus on improving the rigour of clinical practice guideline (CPG) development practices. However, few empirical studies on CPG development have been conducted in low- and middle-income countries. This paper explores national stakeholders’ perceptions of processes informing CPG development for primary healthcare in South Africa, focusing on both their aspirations and views of what is actually occurring.

**Methods:**

A qualitative study design was employed including individual interviews with 37 South African primary care CPG development role-players. Participants represented various disciplines, sectors and provinces. The data were analysed through thematic analysis and an interpretivist conceptual framework.

**Results:**

Strongly reflecting current international standards, participants identified six ‘aspirational’ processes that they thought should inform South African CPG development, as follows: (1) evidence; (2) stakeholder consultation; (3) transparency; (4) management of interests; (5) communication/co-ordination between CPG development groups; and (6) fit-for-context. While perceptions of a transition towards more robust processes was common, CPG development was seen to face ongoing challenges with regards to all six aspirational processes. Many challenges were attributed to inadequate financial and human resources, which were perceived to hinder capacity to undertake the necessary methodological work, respond to stakeholders’ feedback, and document and share decision-making processes. Challenges were also linked to a complex web of politics, power and interests. The CPG development arena was described as saturated with personal and financial interests, groups competing for authority over specific territories and unequal power dynamics which favour those with the time, resources and authority to make contributions. These were all perceived to affect efforts for transparency, collaboration and inclusivity in CPG development.

**Conclusion:**

While there is strong commitment amongst national stakeholders to advance CPG development processes, a mix of values, politics, power and capacity constraints pose significant challenges. Contrasting perspectives regarding managing interests and how best to adapt to within-country contexts requires further exploration. Dedicated resources for CPG development, standardised systems for managing conflicting interests, and the development of a political environment that fosters collaboration and more equitable inclusion within and between CPG development groups are needed. These initiatives may enhance CPG quality and acceptability, with associated positive impact on patient care.

## Background

Clinical practice guidelines (CPGs) have become a familiar tool in policy and clinical practice. CPGs have a range of purposes, intended to improve the efficiency and cost-effectiveness of health system utilisation and to decrease preventable mistakes [1]. They generally include statements of expected practice, benchmarks against which individuals may audit and potentially improve their practices, and guidance regarding undertaking given tasks [1]. CPGs were historically built mostly on expert opinion, which included variable (and often selective) reference to research evidence [2–4]. However, over the last decade there has been increased focus on improving the quality of CPGs and the methodological rigour of their development [5]. Globally, health system pressures are increasingly demanding that resources are effectively allocated and based on research evidence of ‘what works’ [6]. Within this context, there is growing recognition that high quality, evidence-informed CPGs can serve as practical vehicles for meeting these demands and reducing the gap between evidence, policy and best practice [1, 7].

This maturing CPG development culture is evidenced by the various recent attempts made by well-credentialed international collaborations to standardise and improve the credibility of CPG development practices [8]. Between 2011 and 2013, three sets of standards were independently proposed to assist CPG developers in addressing key issues of quality, as follows: the Institute of Medicine introduced 8 standards for guideline development [9], the Guidelines International Network produced 11 relatively similar standards [10], and McMaster University compiled a checklist of 18 topics and 146 items to guide developers [1]. Concurrently, two checklists (the AGREE II instrument [11] and iCAHE guideline quality checklist [12]) were developed, both providing tools to evaluate the process of CPG development and the quality of its reporting. While there are differences between these standards, they are unified in advocating for CPG development to be guided by transparently constructed and evidence-informed approaches that have a clear and applicable scope and are integrated with stakeholder consultation [8].

Despite a growing knowledge industry centred on CPG development, little is currently known about this topic in low-resource settings generally and in sub-Saharan Africa (SSA) in particular [13–15]. While numerous studies in high-income countries (HICs) have investigated the quality of CPGs and their development methodologies in a variety of settings [16], very few of these studies have been conducted in SSA. For example, a systematic review evaluating 42 guideline appraisal studies, including 626 guidelines published between 1988 and 2007, found only 6 guidelines across the studies were from Africa [17]. Nevertheless, the situation is beginning to change, with the topic receiving more empirical attention in SSA over the last 5 years. For example, recent quantitative reviews have evaluated the quality of CPGs for priority diseases in SSA generally [13] and in South Africa specifically [18]. Similarly, certain qualitative studies have provided analyses of the development processes of guidelines for eclampsia treatment and malaria control in 3 SSA countries [19, 20], as well as for maternal health [21], lay health workers [22] and primary care [23] in South Africa.

This body of research in SSA has revealed many similar shortcomings in the quality of CPGs and their development as those identified in studies in HICs, including with regards to their methodological rigour, editorial independence and applicability to local practice [13, 18]. However, it has also shed light on certain unique, context-specific challenges facing CPG development in the region. For example, complex political environments and interests, bureaucratic processes and budget struggles, a lack of locally relevant evidence, and limited skills have all been shown to hinder CPG development in SSA [19–23]. Taken together, the findings from this small body of research suggests that more knowledge is needed on the specific circumstances, processes and priorities underpinning CPG development in different SSA settings, and the factors that could improve their construction. This knowledge will help pave the way for better focused, locally tailored and effective interventions to improve CPG quality and development, and associated positive impact on healthcare practices and outcomes in the region.

Against this backdrop, the aim of the current study was to explore national stakeholders’ perceptions of current CPG development activity for primary healthcare (PHC) in South Africa. More specifically, it sought to investigate their perceptions of the processes that should inform national CPG development, and their sense of what is actually occurring. This study is a sub-study of a broader qualitative study that provided an overview of the current landscape of PHC CPG activity in the country [23]. This sub-study explores the issue of CPG development in more depth, with a focus on the perspectives of stakeholders operating at the national level. Both the larger study and this sub-study form part of the South African Guidelines Excellence (SAGE) project, which aims to understand and improve the development, adaptation, implementation and use of PHC CPGs in South Africa [24].

### Research context

CPGs have been part of South African clinical practice for many decades. Formal national CPG processes were put in place in the mid-90s in a bid to address historical inequity in health service delivery in the nine provinces. South Africa’s National Department of Health (NDoH) currently spearheads several primary care guideline programmes, including condition-specific guidelines (e.g. malaria, HIV, tuberculosis) and the Essential Medicines Programme, which develops comprehensive Standard Treatment Guidelines for rational prescription at all levels of care in an equitable, cost-effective manner. Additionally, academic departments and professional societies develop CPGs, addressing gaps in what is available from NDoH.

## Methods

This study adopted a qualitative approach to understand the phenomena under investigation as experienced and perceived by the actors involved. The methods have been described in detail elsewhere [23], and thus a brief summary is provided here, together with a more detailed description of the analysis methods used in this paper.

### Participants and data collection

The sample included 37 participants from a range of disciplines, stakeholder groups and provinces in the country (Table 1). Participants were approached in their capacity as contributing to national South African CPG development. Most were from academic contexts and providing expert input to guideline panels, many had been involved with this for more than 15 years. Besides the government players, the other participants usually held multiple roles, such that, in addition to CPG development, some were responsible for research, clinical teaching and senior management roles in academic or private sector contexts. Only one government member had a specific role in CPG implementation. Data collection comprised in-depth individual interviews, conducted together by two interviewers per interview. The interviews were based on a semi-structured interview guide, including open-ended questions and tailoring to the experiences of each specific interviewee. Key themes explored included players involved in CPG activity, CPG nomenclature and terminology, as well as the processes, contexts and values underpinning CPG development, adaption, contextualisation, implementation and use in South Africa.

**Table 1:** Description of stakeholders sampled (*n* = 37) [23]

Background discipline	Medicine (*n* = 19), pharmacy (*n* = 5), nursing (*n* = 4), allied health (*n* = 3), dentistry (*n* = 1), nutrition (*n* = 2), non-clinical managers (*n* = 3)
Sectors and stakeholder groups	National (*n* = 10) and Provincial Department of Health (*n* = 2), Professional Societies (*n* = 6), Private sector (pharmaceutical *n* = 1 and medical schemes *n* = 2), academia (*n* = 14), non-governmental organisations (*n* = 2)
Provinces represented	Eastern Cape (*n* = 1), Gauteng (*n* = 16), Kwazulu-Natal (*n* = 3), Western Cape (*n* = 17)

### Data analysis

The data for this paper were analysed through thematic analysis [25] and the lens of an interpretivist conceptual framework [26].

The larger SAGE project previously developed a list of conceptual components (‘open coding’), guided by the research objectives of the SAGE project. After immersion in the transcripts, codes related to ‘CPG development’ within the SAGE coding framework were used to code the transcripts through multiple line-by-line readings and with the aid of Nvivo10, a software programme that aids in the management of qualitative data. Additional or revised codes were developed iteratively as determined by the data and added to the coding framework. Initial and revised codes were then collated into potential themes and produced into an overall ‘thematic map’ to guide further analyses. Using the thematic map, the themes related to the processes of developing CPGs were identified, named and extracted. For each theme, the focus was on capturing how the participants were making sense of CPG development, and the values and concerns they attached to this issue. While we focused on identifying common themes, we also paid attention to the presence of potential diversities in participants’ perspectives. Further analyses were then undertaken to check if these ‘fitted’ in relation to the coded extracts, to refine the specifics of each theme and their relationships with each other, and to contextualise these themes within the other emerging topics in the dataset.

Throughout the analysis process, we attempted to adhere to the methodological principle of reflexivity [27]. At regular intervals SC, TK and KD jointly discussed and further unpacked the emergent themes. Along with facilitating verification, validation and refinement of ideas, these discussions also provided opportunities for the researchers’ interests and taken-for-granted assumptions to surface and subsequently be examined. Such reflexivity was facilitated further by the different roles the researchers occupied in relation to the research, an awareness of which provided for an illuminating interplay of emic–etic viewpoints. TK, KD and AA had jointly conducted most interviews, both had prior knowledge of many participants and TK is involved with CPG activities within academic and government settings. KD and AA are social scientists, and had been involved from project inception with planning, interviews and initial analysis. KD has been involved with health policy analysis in another project. AA straddles social science and quantitative research in her role at the Cochrane Centre. SC became involved in the research only at the analysis stage and had little prior knowledge of the local CPG landscape. SC’s a priori unfamiliarity with the interview content enabled the data to be explored openly and with a ‘fresh’ perspective. Moreover, the interaction between researchers with both ‘distance’ and ‘closeness’ allowed for previous understandings to be opened-up and questioned, and for our own positioning and associated shaping of the research process and outcomes to be critically reflected upon.

## Results

The analysis revealed six ‘aspirational’ processes that participants perceived to be most important when developing CPGs for PHC in South Africa, namely (1) evidence, (2) stakeholder consultation, (3) transparency, (4) management of interests, (5) communication/co-ordination between different CPG development groups, and (6) fit-for-context. We describe each process separately, unpacking both participants’ aspirations and their views of the extent to which each process is, in reality, underpinning the development of CPGs. To preserve anonymity, certain phrases have been removed from quotations and replaced with alternative text in square brackets.

### Evidence

The concept of an ‘evidence-based approach’ featured prominently in participants’ narratives. An overwhelming majority of participants, across stakeholder groups, strongly emphasised that CPGs should be driven by scientific evidence on the effectiveness, safety and cost-efficiency of a clinical process or treatment. The notion that CPGs “*should be evidence-informed*” (INT33), “*scientifically rigorous*” (INT14) and “*guided by the current evidence-base*” (INT25) was widespread, or as one government participant succinctly stated: “*The department stand is that whatever policies or guidelines we develop are evidence based*” (INT22).

A common sentiment, particularly amongst academic and government participants, was that the development processes of many national CPGs have evolved over time, increasingly being informed by a more robust evidence-informed approach. Many CPGs were described as “*coming a long way*” (INT06) or having “*over time, become more and more evidence based*” (INT4). Whilst perceptions of progress were common, the need for further improvement was also communicated. More specifically, many participants expressed reservations about certain CPGs in the country, and the extent to which they are being guided by an evidence-based approach.

Various reasons were provided for the inadequacies participants saw with regards to the certain CPGs’ use of evidence. Here, a lack of dedicated time and funding, as well a scarcity of skills for quality CPG development emerged as overriding themes. The interviews were saturated with accounts of how those involved with CPG development are often doing it voluntarily and afterhours, and there is thus limited capacity to undertake the necessary methodological work:

“*People are stretched… we all have full time jobs and we’re doing it, not for money, not for kudos… so you can’t expect the kind of rigor that you’d like to see*”

(INT04, Academic)

Along with limited time and funding, many participants also highlighted how there is a dearth of skills in the country for synthesising and incorporating evidence in CPGs. Many spoke about the “*lack of competent people who are able to do this kind of work at a national level*” (INT03) and that “*the distribution of people with skills in evidence-based medicine… is quite a problem in the country*” (INT15).

The shortages of skills in evidence-based medicine within the NDoH, in particular, emerged as a key issue amongst government participants. Many attributed this deficiency to what they perceived as inadequate, or even non-existent, in-house training. As one participant put it succinctly:

“*We have also not been trained on the processes that need to be followed*”

(INT37, NDoH)

Similarly, another government official replied, when asked whether he received training in evidence-based medicine:

“*No training, no training! You learn on the job… when you see courses being offered… in most cases it’s out of your own pocket because it’s outside of the HR development planning process*”

(INT22, NDoH)

### Stakeholder consultation

There was considerable agreement amongst participants and across stakeholder groups that widespread stakeholder consultation also needs to form an essential part of CPG development. Many participants spoke at length about why this is critical. Along with serving as an important peer-review mechanism, widespread consultation was seen as essential for facilitating ‘buy-in’:

“*I think a key for any guideline is inclusivity. Because if you want people to embrace your guideline, it’s much easier if they were part of it than if you just thrust it upon them*”

(IN19, Professional society member)

Several participants felt that it is particularly important to include the end-users of CPGs in consultation processes to ensure that guidelines are “*practical… at implementation level*” (IN32), are “*acceptable to those implementing them*” (INT22) and “*so nurses understand what you want to say*” (INT05).

Many participants spoke about their own CPG development processes as involving stakeholder consultation, one which they perceived to be fairly wide and extensive. Participants spoke about “*our strategy of wide consultation*” (IN22), that “*we are fairly meticulous about circulating our guidelines to all organisations concerned*” (INT17) and that, ultimately, “*Anybody who is on the ground, who feel they have something to contribute here, they will*” (INT16).

While there was a tendency amongst participants to describe their own consultation processes in relatively positive terms, a more complex picture of stakeholder engagement in CPG development also surfaced. This emerged most prominently when participants talked about their experiences with the development processes of other CPG development groups. This also materialised in certain participants’ narratives of their own consultation processes, where certain reservations were revealed.

Many participants spoke about various other CPG development groups as comprising “*a very non-consultative process*” (INT18) or as being “*an authoritative entity, who never… considers clinicians’ input*” (INT17). Others described the inherent unresponsiveness of certain CPG groups, and their inadequacies in responding to people’s input. As one participant said:

“*The sense that we’ve had with all the people we’ve engaged with is that you will send a lot of feedback to the* [particular CPG development group] *but you’ll get no formal response to any of your feedback*”

(INT03, Academic)

This failure to provide adequate feedback was recognised by certain participants with regards to their own consultation processes. Many explained how there actually is a very rigorous process for considering and incorporating stakeholder feedback, and yet due to capacity constraints, they are limited in their ability to adequately respond. As one participant indicated, acknowledging that this is a problem:

“*People will give a comment, but when the book is published they see that their comment hasn’t been incorporated… it’s not that the committee didn’t consider the comment… but because of capacity constraints, we can’t respond to each and every comment… but we’ve got to ask ourselves: how do we make it more publicly available that we have looked at your comment… without responding to each and every person on each and every point?*”

(INT16, NDoH)

Certain participants conveyed other reservations with regards to their own consultation processes. Some questioned the level of inclusivity of their engagement processes, highlighting the problems they have engaging with particular groups. For example, many professional society participants alluded to the struggles they encounter consulting with government:

“*I think the process should be more inclusive… that is certainly the weakness of our current situation. But engaging with government is an extremely difficult process… there are very, serious barriers of communication with government*”

(INT18, Professional society member)

Many other professional society participants shared this participant’s view, providing similar accounts of how “*communication with the DoH has been shocking*” (INT19), and how “*there should be an easier way for us to engage government*” (INT14).

Certain government participants alluded to the difficulties they experience around engaging with various groups. Some felt that particular provinces, other than the Western Cape and Gauteng, are hard to engage with and ultimately remain weak in their participation:

“*I think there are weaknesses within the consultations at provincial level… in a province like Western Cape, it is done widely, but in other provinces, not really that much*”

(INT22, NDoH)

Other government officials felt that the end-users of CPGs are another specific group that they have found hard to reach and are unsatisfactorily consulted, as reflected by this participant’s comment:

“*I don’t think we have found a better… mechanism of really engaging the people who are… at the frontline of implementation… I think a lot of programmes struggle with what will be the best way of engaging the end users*”

(INT36, NDoH)

Those participants who expressed uncertainties about their own stakeholder consultations suggested various aspects of the engagement process that might limit its inclusivity. For example, some indicated that the process tends to favour those who have the capacity to provide written feedback and the ability to use the internet, as depicted in the following two statements:

“*So* [a particular CPG development group] *have… collated a whole lot of emails and they send it out and also make it available on the website… but it just depends sometimes though, like nurses in rural areas do not have the capacity to connect online*”

(INT33, Academic)

“*The same usual suspects will give their comments because that’s their comfortable way of engaging… but there are other people who engage differently… maybe they don’t want to write something so they may need a different strategy*”

(INT16, NDoH)

Relatedly, other participants suggested that the time given to stakeholders to provide feedback is insufficient, and may be an additional barrier to more widespread and inclusive consultation:

“*The consultation process doesn’t always look valid because there wasn’t given time to comment*”

(INT14, Professional society member)

### Transparency

It was widely suggested that CPG development also needs to be guided by a clear, transparent process so people can understand decision-making processes. Many participants spoke about the fact that “*people need to see the validity of the process*” (INT01) and that “*fair transparency is a critical component of guideline development*” (INT23), or as one participant stated:

“*I think the most important thing is that… we have to have a very transparent, clear process… a level of transparency that… someone can understand why decision were made*”

(INT08, Professional society member)

The dominant view expressed by participants in all the stakeholder groups was that CPG development in the country tends to lack sufficient transparency. Descriptions were commonplace about how CPG construction processes are “*very untransparent… completely opaque to everybody*” (INT14), “*a complete mystery*” (INT3), or as described by one participant:

“*I have issues with the* [particular CPG development group] *not being transparent… what we need is to make that process visible, because it’s actually, they have terms of reference, they have criteria, they go through a very evidence-based process*”

(INT30, Academic)

Like this participant, many other participants conveyed a sense of trust in the rigour of various CPG development processes, and yet perceived there to be a significant gap in the documentation and sharing of the logic behind the decisions. Many suggested that there is a need for greater communication about exactly how the process unfolded, so that people can better appreciate the credibility of CPGs:

“*I think the communication… there’s a lot of misconceptions… but just talking to people and telling them, okay, this is how we do it, then they get the insights… that there’s a rigorous process*”

(INT16, NDoH)

Not many participants provided reasons for why the process is not as transparent as it should be, despite probing by interviewers. The few that did reflect on this issue suggested, once again, that limited time and funding was the cause, with stakeholders lacking the capacity to adequately record and elucidate decision-making processes:

“*So there is an awful lot of work going on… but not a lot of capacity to engage in a very clearly documented and open process… but we do need to consider what the best means is of documenting evidence and then sharing that evidence in order to get more buy-in*”

(INT21, Academic)

Given that few participants provided details on why transparency is an issue, it is unclear whether this view is shared by others.

### Management of interests

A few participants stated explicitly that the declaration and management of interests need to form a key aspect of CPG development, so that decision-making processes are not influenced by inappropriate forces:

“*Your governance has to be clear… people have to know… what interest they need to declare, what information they need to keep confidential… and the reason… is because we don’t want undue influence on our decision-making processes*”

(INT16, NDoH)

Although not overtly stated in most cases, many participants clearly conveyed the view that vested interests need to be considered and managed when producing CPGs. The interviews were replete with descriptions of people’s conflicts of interests and the role these are playing in CPG development. Participants spoke about these interests as being both personal/intellectual and financial. In terms of the former, many described in detail the “*personal agendas*” (INT5), “*little hobby horses*” (INT6) and “*vested interests*” (INT14) people involved with CPG development across the board have:

“*There’s a whole lot of politics… we have all these competing interests… even us sitting at the university… we have these different groups that have their own agenda… to defend their turf*”

(INT25, Academic)

More specifically, many participants described how individuals developing CPGs may be involved with specific programmes or research projects, and often push for guidelines to incorporate these. As articulated by this participant:

“*There’s a lot of individuals or research institutions pushing their own agendas… like those on drug development, clinical trials… the expectation is that you would change your policy based on that… and it creates problems for us in terms of determining what should be in the guidelines… we’re put under pressure*”

(INT22, NDoH)

Numerous participants provided analogous accounts, similarly highlighting the problems they have around managing personal interests. Certain participants also expressed uncertainty about how these agendas can and should be managed, as communicated by this participant:

“*We talked about this… how we probably need to also disclose grant conflicts of interest because if you’re sitting on a study that is, you know, if you change* [the policy], *your study is not going to continue, right?… But, then everybody has some sort of bias, so I don’t know what the ultimate answer is, like how do you make this so completely transparent*”

(INT08, Professional society member)

In addition to interests of a personal or intellectual nature, many participants were also particularly concerned about financial interests and the fact that a “*massive amount*” of guidelines are “*driven by industry*” (INT20). As aptly revealed by this remark:

“*My colleagues… they don’t see the harm if industry comes and does this. You know, it’s so insidious… they’re doing subliminal advertising, and people don’t get how that can influence how you make a decision*”

(INT16, NDoH)

When talking about the pervasiveness of financial interests, many participants were particularly worried about professional society groups in this regard. Comments about such groups having “*lots of apparent influence of industry*” (INT21) and “*being influenced tremendously by industry*” (INT18) were ubiquitous, along with descriptions of how professional society CPGs are “*essentially drug company driven*” (INT19). Many participants also questioned the sufficiency of the extent to which the financial interests operating amongst professional societies are being managed:

“*In many instances, if not most instances, there is no process to deal with potential conflicts of interest*”

(INT15, NDoH)

Certain professional society participants themselves expressed analogous concerns, with some conveying similar apprehensions about the presence and inadequate management of financial interests within their own societies. As one member acknowledged:

“*It depends on the integrity of the individual… you know, I think it’s very glib now, the declaration of conflict of interest. It goes up in the first slide and you don’t even see it. Here’s my title and here’s my conflict of interest. It’s very glib*”

(INT18, Professional society member)

In a similar manner, when asked directly about his experiences of conflict of interests within his own society, another participant explained:

“*I’m trying to remember… whether we actually had to declare a conflict of interest. I don’t think we did*”

(INT19, Professional society member)

This participant went on to articulate why conflicts were not declared within his society, outlining some of the difficulties in this regard. According to him, it would be “*too numerous*” as “*everyone is going to have to have received funding from someone for something*”*.* He explained that “*all those would all have to be declared … which would take the first four pages of the guideline*”*.* He indicated further that “*if you try and put too many rules in place*”, you will ultimately create a hindrance to CPG development, or as he put it: “*It then trumps people’s desire to actually do the guidelines*”.

Thus, while most participants felt that management of interests is a key activity for CPG development, certain stakeholders had a different perspective. As with the participant above, some questioned the value as well as the practicality of declaring and managing conflicts of interests.

### Communication and co-ordination between CPG development groups

Various participants stressed the importance of communication and co-ordination between different CPG development groups, suggesting that “*those writing guidelines must speak to each other*” (INT37) and that “*there’s got to be absolute linkage between programme guidelines*” (INT27). This was identified as essential for ensuring “*harmony between guidelines*” (INT03) and that “*we don’t give confusing messages to practitioners*” (INT15). Many participants felt that it is particularly important for CPG developers to communicate with the Essential Drug List (EDL) committee, as exemplified by this comment:

“*If I was redesigning the system, I would have a… process for guideline development that has a clearing house effect that goes through the EDL, who then issues it*”

(INT14, Professional society member)

There was much consensus amongst the participants that, in reality, communication and coordination between different CPG development groups is noticeably absent. The general picture that emerged was one of fragmentation, whereby a diverse range of groups are developing CPGs relatively independently of each other. Many described this disconnect as occurring between the private and public sectors:

“*Private, they do their own thing, only… where they don’t have a choice… or where they absolutely don’t know, only then do they then refer to the other guidelines*”

(INT06, NDoH)

Other participants spoke about divisions within the NDoH and the lack of communication across government departments. CPG development processes within the NDoH were referred to as “*siloed in a way that there’s not really good communication*” (INT08), “*disjointed pockets of activities*” (INT15), or as one participant proposed:

“*There is a two-parallel process from the department of health, and the one side is the formal process and on the other side you’ve got stroke management, malaria management, HIV management… so all are little silos inside other silos*”

(INT20, NDoH)

At the same time, many participants felt that some, but not all, CPG development groups are communicating with the EDL, or as two participants put it “*communication is stronger with some programmes than with others*” (INT37) and “*often programmes haven’t checked the EDL*” (INT20). This view was shared by a member of the EDL committee who, when asked whether CPGs are being circulated through the EDL, responded:

“*It doesn’t happen with all national departments. So* [particular government department], *yes, but I’m still struggling to get* [particular government department] *to send their guidelines to us for peer review*”

(INT17, Academic)

This lack of communication between CPG developers was perceived to result in the replication of guidelines and a duplication of work, or in the words of one participant: “*discrete pockets of people reviewing the same data*” (INT11). It was thought to also give rise to contradictions between CPGs, with “*a whole host of conflicting recommendations across the different guidelines*” (INT03) and guidelines that “*don’t fit together… to make a coherent whole*” (INT02).

Some participants attributed this situation to a matter of timing, suggesting that CPG inconsistencies are related to the fact that different CPGs are developed and updated “*according to different schedules*” (INT37) that are “*not always in sync*” (INT15). However, other participants conceived the problem to be of a more political nature. That is, it was suggested that the difficulties stem from the complex relations of power and control that exist within the CPG development arena in the country, as one participant proposed:

“*There are lots of interest groups competing for control of this, and that’s why partly it hasn’t been cohesive… The department of health, because they are in the HIV field, I think wants to keep some of that to themselves. The TB people wanted the TB to themselves… there’s just lots of competing interests around these things*.”

(INT14, Professional society member)

### Fit-for-context

A final issue that featured prominently in the interviews was the need for CPGs to be contextually relevant, and thus the necessity that CPG developers think about “*what is suitable for our context*” (INT01) and “*is this relevant to our situation*” (INT37), or as specifically stated:

“*We feel guidelines need to be relevant to South Africa, I mean, our situations are different and our cost constraints are different, and it has to be relevant*”

(INT18, Professional society member)

When describing CPG development processes, there was considerable agreement amongst participant groups that most CPGs in the country draw heavily on international guidelines and what is being recommended globally, particularly by WHO:

“*In most cases guidelines are guided by the WHO recommendations*”

(INT22, NDoH)

Although there was widespread consensus that CPGs in South Africa are usually based on what is being done and advocated for internationally, there were divergent views amongst the participants about the use of this approach. Some were critical of this tendency, suggesting that we should not be relying on other sources to do the methodological work of CPG development:

“*I would say there’s an over reliance on other guidelines without looking at the primary evidence*”

(INT37, NDoH)

Other participants expressed frustration with the dominance of global discourse, and the pressures they feel to conform to these. This was aptly conveyed by one participant who lamented:

“*WHO is a really sore point with a lot of our experts because they’re writing policy for Africa… I mean we do not lack technical expertise… and we have the evidence… I think our experts should be the ones driving the decisions*”

(INT14, Professional society member)

In contrast to these perspectives, other participants supported the widespread use of international guidelines when developing local CPGs. Here, the common view was that it is unwarranted to repeat the work already done by other, well-respected organisations:

“*It’s not necessarily developing all the guidelines from scratch… because if it’s there, why reinvent the wheel*”

(INT25, Academic)

Many participants shared this view, yet suggested further that international CPGs should be used but a process of contextualisation should ensure the “*critical appraisal of international guideline*” and “*local adaptation*” of these so they suit out local circumstances (INT37). As two professional society participants explained:

“*What I have been pushing for a lot is to say, take the WHO guidelines… and then adapt them to South Africa*”

(INT14, Professional society member)

“*What we want to do,* [which] *we haven’t always historically done… is we need to take WHO as the starting point, the baseline and then adapt from there*”

(INT08, Professional society member)

As suggested by these two participants, and sharing the views of other participants, the process of adapting international CPGs is currently limited, something which was perceived to be a key area for improvement. However, there were some participants who indicated that they do undertake such a process, stating, for example, that “*we use international ones and then adapt for this context*” (INT25) or that “*a lot of it is based on WHO recommendations and then we decide whether it is suitable for our context*” (INT30). Despite considerable probing, details of exactly how this process of appraisal and adaptation is undertaken was difficult to gage, with participants providing little information in this regard.

While the importance of local relevance was widely emphasised, the majority of participants indicated, simultaneously, that CPGs should not be adapted for contextual difference within South Africa. With one exception, all participants emphasised strongly that “*we don’t want disparity between provinces*” (NT16) and that “*you can’t have a different standard for Limpopo* [poorly resourced province] *and for the Western Cape* [well-resourced province]” (INT04). In justifying this view, many participants explained how the overriding objective of post-1994 CPGs is to promote justice and ensure everyone has access to the same standard of care, so as to redress the historical inequities instigated by the apartheid regime. Thus, according to them, adapting CPGs for inter-provincial contextual differences would be going against the very socio-political role CPGs are meant to play in the country. However, one participant, a government official from Kwa-Zulu Natal, had a contrasting perspective, explaining how South African provinces have vastly different resources, cultures and infrastructure, and that a failure to accommodate these diversities could have dire consequences:

“*Our needs might not necessarily be the exact same as other provinces… and sometimes you find that a national guideline is… not tailored for the province… and the moment you say you must without considering the situation in the province it will make a situation worse, it won’t help us*”

(INT32, NDoH)

## Discussion

This paper explored national stakeholder participants’ perceptions of the processes informing CPG development for PHC in South Africa, focusing on both their aspirations and sense of what is occurring ‘in reality’. While the analysis sought to identify common themes, it also paid careful attention to potential divergences in perspective.

The findings revealed considerable agreement amongst participants about the processes that should inform CPG development in the country. While there were differences in the relative importance given to each of the six aspirational processes identified, all were highlighted as important by the 37 participants. These processes strongly reflect current global standards regarding guideline development, and their emphasis on inter alia use of evidence, stakeholder involvement, transparency, applicability and editorial independence/managing conflict of interest [1, 9, 10]. The importance of ‘evidence’ was a particularly prominent theme. This widespread culture of evidence-based medicine amongst our respondents, and similarly revealed in other policy development studies in South Africa [21, 28], stands in sharp contrast to the literature describing a lack of access to and awareness of evidence in many low- and middle-income countries [29, 30].

Another theme that featured strongly in the participants’ narratives was the importance of CPGs to be ‘fit for context’. While there were divergent views about the pervasive use of international guidelines, and evident tensions around whether CPGs should accommodate provincial differences, the imperative to consider ‘context’ when developing CPGs was a common viewpoint. There is growing interest within the international CPG methodology literature in ‘contextualisation’, with various frameworks recently developed to guide the adaption of CPGs developed in one country to other settings [31, 32]. However, these approaches tend to be designed for the reconfiguration of CPGs to new, but contextually similar, settings [5, 33]. They thus provide little guidance on how CPGs might be transferred across settings with different healthcare policies and contexts. The Filipino CPG implementation project [33] and Practical Approach to Lung Health in South Africa initiative [34] are examples of the few attempts that have been made to develop practical approaches for contextualising CPGs developed in HICs to low- and middle-income countries. More of these kinds of initiatives are clearly needed, as evidenced by participants’ uncertainty in this present study around how to adapt CPGs developed in HICs for effective use in South Africa.

A particularly noteworthy finding from this study was the chasm between participants’ aspirations of how things ‘should be’ and their views of how things are ‘in reality’. While many spoke about a transition towards more robust processes, the general view was that CPG development still faces significant challenges with regards to all six aspirational processes highlighted. Across all six thematic areas, there were suggestions for how best to bridge the gap between what is, and what should be happening, in CPG development processes; we explore these suggestions in another paper [23]. Concurring with other guideline development research in in SSA [19–23], many of the problems identified were attributed to a lack of financial and human capacity. More specifically, the paucity of dedicated time, funding and skills for CPG development was perceived to hinder the methodological work of synthesising evidence, effectively responding to stakeholders’ feedback, and transparently documenting and sharing decision-making processes. A lack of resources is not a unique issue affecting CPG development in South Africa. Despite the introduction of key health policy reforms, human and financial resource challenges continue to hamper progress in all areas of healthcare delivery [35]. The findings in this paper suggest that, like in other areas of healthcare, there is a need for greater dedicated resources for CPG development to build capacity and support for the delivery of high quality CPGs in South Africa.

In addition to resource limitations, our findings suggested that current challenges facing CPG development are also intimately linked with the complex web of politics and power operating within the CPG arena in the country. For example, the fragmentation within government programmes and between public and private sectors was attributed, in part, to issues of control and ownership. Different groups were described as interested in maintaining their authority over specific territories, thus sabotaging efforts for collaborative CPG development work. As proposed elsewhere [23] and supported by this study, a possible way forward would be for South Africa to have a centrally coordinated CPG unit with buy-in from and communication between public and private stakeholders. It would be important for this unit to help foster a political environment that promotes collaboration and integration between different CPG development groups.

Simultaneously, the CPG arena was seen to be saturated with personal, financial and political vested interests, and lacking processes for reporting and managing these. The issue of conflicting interests is not unique to South Africa. Globally, many guideline development groups fail to adequately disclose and manage conflicts of interests [36, 37]. Most certainly, guideline development is never neutral, and is inevitably “*a social as well as technical process*” that “*necessarily reflect*[s] *value judgments*” [38]. However, clear procedures for the documentation and management of interests, including financial relationships and sources, are essential. The varying perspectives participants in this study conveyed around if and how conflicting interests can and should be managed suggests that this is a complex issue in the country. Further research on this topic in South Africa is therefore needed, and substantial stakeholder input and buy-in will be required if we are to move from how interests have been managed to how they should be.

Finally, challenges pertaining to stakeholder engagement were also described as related to power dynamics in the country. The nature of CPG consultation was perceived to favour those individuals and groups who have the time, resources and capacity to provide input. As such, and as reported in other studies in South Africa [22, 39], the views of end-users of CPGs, ‘at the coal face’ of service delivery remain inadequately incorporated. As suggested by participants in this present study, and similarly highlighted elsewhere [40, 41], a failure to include the perspectives of those who will be implementing CPGs could have a dire impact on their effective uptake and use. A noticeable absence in the stakeholders’ narratives in this study pertains to the issue of patient involvement. Internationally, patient engagement is now recognised as an important component of CPG development to ensure the production of more patient-centred and trustworthy guidelines [42, 43]. The silence around this topic in this study suggests that the involvement of patients in CPG development clearly requires more attention in South Africa.

Additionally, it emerged that the process of stakeholder engagement tends to marginalise the opinions of individuals and groups located in the more resource-limited provinces in the country. This is indeed further supported by the fact that, despite our attempts to include national stakeholders from all provinces in the country in this study, our final sample was dominated by participants from Gauteng and Western Cape Provinces, two of the country’s most well-resourced provinces. Read in conjunction with the study findings, this sample bias is likely to reflect the reality of skewed power dynamics in CPG development in South Africa, where many who lead national knowledge production, and are therefore able to contribute their time to the process, may be based in the Western Cape and Gauteng. Ultimately, all of this suggests that CPG development in South Africa needs to develop more innovative strategies for better reaching and including the voices of those across professional hierarchies and provinces in the country, as well patients and healthcare consumers.

### Study strengths and limitations

The strengths and limitations of the broader study in which this sub-study is embedded, have been described in detail elsewhere [23]. With regards to this sub-study specifically, we have focused on the personal accounts and experiences of respondents. A strength of this approach is that the data represents the perspectives of actors engaged directly with CPG activity in South Africa and therefore provide valuable insights into the thinking behind CPG development processes. However, we recognise that such accounts are inevitably influenced by respondents’ position at time of event, their position at the time of being interviewed, their relationship with the researchers and their memory of particular events and processes [44]. Our final sample was dominated by participants from Gauteng and Western Cape Provinces, and thus the views and perspectives of stakeholders who may be involved in CPG development from other Provinces in the country might not have been sufficiently reflected. At the same time, we did not speak to patient representative groups or patients, as it emerged during our stakeholder ‘mapping exercise’ that these individuals and groups are currently limited in their involvement with CPG development in the country. Further research in CPG development in South Africa, which tries to reach more ‘marginalised’ stakeholders, including patients and patient representative groups, is needed.

Furthermore, and as noted in the previous paper [23], the lead researcher, who was present at most interviews, has experience in evidence synthesis for CPGs and teaching about CPGs. As she may be known to some interviewees as an advocate for evidence-based healthcare, this may introduce response bias. Therefore, we ensured that at least two interviewers were present, including one who was not engaged in CPG activities, with the intention to create more distance and, as much as possible, objectivity. In addition, and as described in the methods section, the researchers discussed the findings at regular intervals during the analysis process. This helped to verify and refine the emerging themes and provided an opportunity for our presuppositions (and how they may be shaping the analysis) to be identified and critically examined.

While the results of this study need to be generalised with caution, as all qualitative research, we have succeeded in providing in-depth insight into CPG development in South Africa. The results corroborate with and extend the findings from other studies on this topic in SSA and South Africa more specifically. The results also shed light on key factors that might help to improve the development of high-quality CPGs in South Africa, and potentially other countries in the region.

Many of the findings in this study are similar to those previously reported. However, two unique issues that emerged, which have not received much discussion elsewhere, were the complexities around managing conflicting interests and adapting CPGs to within-country contextual differences. Participants in this study held contrasting perspectives about these issues, suggesting the need for further research into these factors and greater discussion regarding how they should be addressed.

## Conclusion

Growing awareness of the important role CPGs can play in healthcare systems in SSA demands increased knowledge about CPG development activity in the region. Focusing on South Africa, this study has shown that, while there is strong commitment amongst national stakeholders to advance guideline development processes, a complex mix of values, politics, power and capacity constraints pose significant challenges in this regard. More dedicated resources for CPG development, together with standardised conflict of interest policies and greater guidance for trans-contextual CPG adaption will help enhance the quality and credibility of CPGs for PHC and have an associated positive impact on patient care in the country. Cultivating a political environment that fosters collaboration, reciprocity and more equitable inclusion within and between different CPG development groups and stakeholders could help reduce duplication of efforts, make better use of limited resources and skills, and help redress historical inequities within the South African healthcare context.

## Abbreviations

CPG: clinical practice guidelines
EDL: Essential Medicines List
HICs: high-income countries
NDoH: National Department of Health
PHC: primary healthcare
SAGE: South African Guidelines Excellence
SSA: sub-Saharan Africa
